# Inhibition of Bone Marrow-Mesenchymal Stem Cell-Induced Carbonic Anhydrase IX Potentiates Chemotherapy Efficacy in Triple-Negative Breast Cancer Cells

**DOI:** 10.3390/cells12020298

**Published:** 2023-01-12

**Authors:** Annachiara Sarnella, Ylenia Ferrara, Sandra Albanese, Daniela Omodei, Laura Cerchia, Giuseppina De Simone, Claudiu T. Supuran, Antonella Zannetti

**Affiliations:** 1 Institute of Biostructures and Bioimaging, CNR, 80145 Naples, Italy; 2 Institute of Experimental Endocrinology and Oncology “G. Salvatore”, CNR, 80131 Naples, Italy; 3 Department of Neurofarba, University of Florence, 50019 Florence, Italy

**Keywords:** tumor microenvironment, bone-marrow-mesenchymal stem cells, triple-negative breast cancer, carbonic anhydrase IX

## Abstract

Conventional chemotherapy represents the main systemic treatment used for triple-negative breast cancer (TNBC) patients, although many of them develop drug resistance. The hypoxic TME is the crucial driver in the onset of insensitivity to chemotherapy. In this research, we elucidated the role played by bone marrow-derived mesenchymal stem cells (BM-MSCs) in reducing cisplatin effects in TNBC. BT-549 and MDA-MB-231 cells, grown under hypoxic conditions in the presence of conditioned medium obtained from BM-MSCs (CM-MSCs), showed a strong cisplatin insensitivity and increased expression levels of carbonic anhydrase IX (CA IX). Therefore, we inhibited CM-MSC-induced CA IX by SLC-0111 to potentiate chemotherapy efficacy in TNBC cells. Our results showed that CM-MSCs under hypoxic conditions caused an increase in the ability of TNBC cells to form vascular structures, migrate and invade Matrigel. Cell treatment with cisplatin plus SLC-0111 was able to block these mechanisms, as well as the signaling pathways underlying them, such as p-AKT, p-ERK, CD44, MMP-2, vimentin, β-catenin, and N-cadherin, more effectively than treatment with single agents. In addition, a significant enhancement of apoptosis assessed by annexin V, caspase-3 expression and activity was also shown. Taken together, our results demonstrated the possibility, through CA IX inhibition, of returning TNBC cells to a more chemosensitive state.

## 1. Introduction

Triple-negative breast cancer (TNBC), which accounts for about 10–15% of all breast cancers, is highly heterogeneous and occurs, in particular, in young women [1]. It is characterized by a lack of estrogen receptors (ER), progesterone receptors (PR) and epidermal growth factor receptor 2 (HER2/ErbB2), therefore, no targeted therapies against these proteins are useful to treat this carcinoma [1].

To date, chemotherapy represents the main systemic therapeutic approach for TNBC, although the early onset of intrinsic or acquired resistance is common, which is the primary cause of recurrence, metastatic spread and poor overall survival of TNBC [2]. Given that TNBC heterogeneity is recognized as a major cause of chemotherapy failure, many attempts have been made to define and categorize it according to the intrinsic characteristics of cancer cells.

However, over the past decade, much evidence has supported the relevance of the role played by the tumor microenvironment (TME) in promoting TNBC heterogeneity and, consequently, response to treatments (extrinsic features) [2]. Many studies focused on the importance of the interaction between tumor cells and multiple components of TME in tumor development, growth, metastasis and treatment response, including drug resistance and relapse, as well as reprogramming of energy metabolism and evading immune suppression [3,4]. Therefore, to better comprehend and predict the behavior of tumors in response to conventional and/or targeted therapies, it is crucial to know the role played by TME, which is an integral part of lesion. In fact, the architecture surrounding the tumor is not static but is subjected to a continuous remodeling in response to the dynamic interplay between tumor cells and stroma [5]. These stromal cells that interact with tumor cells are actively recruited from the other tissues, and once in the TME, they progressively switch from a neutral or anti-tumorigenic role toward a pro-tumorigenic role [6,7]. Mesenchymal stem/stromal cells primed by tumor cells promote tumor malignant features, including epithelial–mesenchymal transition (EMT), propagation of cancer stem cells (CSCs) traits, immunosurveillance, as well as therapeutic resistance [8]. Our previous studies shed light on the role played by bone marrow-derived mesenchymal stem cells (BM-MSCs) in promoting the TNBC metastatic phenotype. In vivo imaging of near-infrared (NIR)-labeled BM-MSCs allowed us to visualize their homing into TME of an orthotopic TNBC animal model and demonstrated their involvement in the enhancement of lung metastases [9]. It was noteworthy that the inhibition of the cross-talk between BM-MSCs and tumor cells by a specific aptamer targeting platelet-derived growth factor receptor β (PDGFR-β) hampered BM-MSC recruitment in TME and the ability of these cells to induce metastases [9]. Recently, we investigated how hypoxic TME impacts on TNBC plasticity by increasing adaptive changes correlated with a more aggressive phenotype such as EMT, vascular mimicry (VM) and stemness and on the involvement of hypoxia-induced pH regulatory enzyme carbonic anhydrase IX (CA IX) in promoting these processes [10]. Furthermore, in this study, we demonstrated, using in silico analysis, that CA IX expression is higher in the TNBC group than in the other breast cancers [10].

CA IX contributes to increases in extracellular acidosis by catalyzing the reversible hydration of carbon dioxide to bicarbonate and proton, thus creating a suitable place where tumor cells acquire characteristics of stemness and the ability to metastasize, resist treatments and escape immunosurveillance [11,12]. We showed that inhibition of CA IX limited CSCs and VM development, as well as hindered the EMT program in TNBC cells [10].

In this study, we investigated the role of BM-MSCs in contributing to reduce cisplatin (Cis-Pt) sensitivity of TNBC cells in a hypoxic TME through overexpression of CA IX and the possibility of reversing this phenomenon by using a sulfonamide CA IX inhibitor, SLC-0111, in Phase Ib/II clinical trials (NCT02215850) [13,14].

## 2. Materials and Methods

### 2.1. Cell Lines and Culture Conditions

Human breast cancer cell lines came from the American Type Culture Collection (ATCC). MDA-MB-231 and BT-549 were grown in RPMI 1640 supplemented with 10% FBS and 1% L-glutamine-penicillin-streptomycin and were maintained in a humidified incubator in 5% CO_2_ at 37 °C. BM-MSCs were purchased from Lonza (PT-2501) and were grown in MSCGM™ Mesenchymal Stem Cell Growth Medium (PT-3001) in a humidified incubator in 5% CO_2_ at 37 °C. BM-MSCs passed the quality inspection conducted by Lonza company using cell viability (more than 75%), adipogenic and osteogenic differentiation (Oil Red O staining and calcium deposition staining) and FACS analysis of cell surface markers (more than 90% were positive for CD29, CD44, CD105 and CD166, and negative for CD14, CD34 and CD45) [15]. To obtain bone marrow-derived mesenchymal stem cells conditioned medium (CM-MSCs), BM-MSCs were grown in medium with 1% FBS for 48 h. The medium was then collected, centrifuged at 1000× *g* for 10 min and filtered through 0.22 μm filters (Millipore, Billerica, MA, USA) before being added to tumor cells [16]. All experiments were performed by growing TNBC cells in normoxic (21% O_2_) and hypoxic conditions (1% O_2_) and in the presence of CM-MSCs. Hypoxia was attained in a modular incubator chamber (Stem Cell, Catalog #27310). The chamber was flooded with the hypoxic gas mixture for 7 min and then sealed and stored in an incubator at 37 °C in 5% CO_2_. CA IX/XII inhibitor SLC-0111, developed in the laboratory of Professor Claudiu T Supuran (NEUROFARBA Department, University of Florence, Florence, Italy) and previously described [17], was used.

### 2.2. Cell Viability Assay

MDA-MB-231 and BT-549 (4.0 × 10^3^ cells/well, 96-well plates) cell viability was assessed with CellTiter 96 AQueous One Solution Cell Proliferation Assay (Promega BioSciences Inc., Fitchburg, WI, USA) using 3-(4,5-dimethylthiazol-2yl)-5-(3-carboxymethoxy-phenyl)-2-(4-sulfophenyl)-2H tetrazolium (MTS) and according to the manufacturer’s instructions. TNBC cell lines were treated with different concentrations of Cis-Pt (0.5, 1, 5, 10 µM) or with SLC-0111 (100 µM) alone or in combination with Cis-Pt at a concentration of 1 µM, under normoxic (21% O_2_) and hypoxic (1% O_2_) conditions in the presence or absence of CM-MSCs for 72 h [18].

### 2.3. Clonogenic Assay

MDA-MB-231 and BT-549 cells (500 cells/well) were seeded into six-well plates. After 24 h they were treated with Cis-Pt (1 µM) and SLC-0111 (100 µM), alone or in combination, at 37 °C in hypoxic conditions and in presence of the CM-MSCs for 10 days. Subsequently, cells were washed with DPBS and were fixed and stained with 0.1% crystal violet in 25% methanol. Following 30 min at RT, culture dishes were washed with DPBS and colonies were photographed [19].

### 2.4. Vascular Mimicry

MDA-MB-231 (1 × 10^4^) and BT-549 (1 × 10^4^) cells were suspended in a 100 μL medium containing 2% FBS in the presence of Cis-Pt (1 µM) and SLC-0111 (100 µM), alone or in combination, and were seeded into 24-well plates, pre-coated with 80 μL/well Matrigel, in hypoxic conditions and in the presence of CM-MSCs for 24 h. Tube formation was analyzed under a phase-contrast microscopy and complete loops were quantified by a macro made with the ImageJ software version 1.52t (National Institutes of Health, USA) [20].

### 2.5. Cell Migration and Invasion Assays

Cell migration and invasion assays were performed using 24-well Boyden chambers (Corning, NY, USA) with inserts of polycarbonate membranes (8 μm pores), coated or not with 50 µL of diluted Matrigel (1:5 in PBS) (Corning, NY, USA). MDA-MB-231 and BT-549 cells (0.5 × 105/well and 1 × 105 /well) were re-suspended in 100 µL of serum-free medium in the presence or absence of Cis-Pt (1 µM) and SLC-0111 (100 µM), alone or in combination, and seeded in the upper chamber in hypoxic conditions and in the presence of CM-MSCs for 24 h (migration) and 72 h (invasion). In addition, 10% FBS was added in the lower chamber as chemoattractant. The non-migrated cells were removed with cotton swabs, whereas the cells that had migrated were visualized by staining the membrane with 0, 1% crystal violet in 25% methanol, as well as the invasive cells. Ten random fields/filter were counted under a phase-contrast microscope (Leica, Wetzlar, Germany) and images were taken with a digital camera (Canon, Tokyo, Japan) attached to the microscope. All experiments were performed at least three times and the results are expressed as the percentage of migrating/invading cells, considering the untreated control sample as 100% [21,22].

### 2.6. Spheroid Formation and 3D Invasion Assays

MDA-MB-231 and BT-549 cells (100 × 10^3^ cells/well) were grown in serum-free DMEM supplemented with B27 (1X), bFGF (20 ng/mL) and EGF (10 ng/mL) in ultra-low attachment 6-multiwell plates (Corning). Cells were incubated at 37 °C with 5% CO_2_ for 7 days. Spheroid formation was analyzed under a phase-contrast microscopy and the size and number of formed spheroids were calculated using ImageJ. In 3D invasion assay, the formed spheroids derived from TNBC cells were embedded with a Matrigel mixture in a ratio 1:1 with their specific medium and were monitored every 24 h, after treatment with Cis-Pt (1 µM) and SLC-0111 (100 µM), alone or in combination. The experiments were performed in the presence of CM-MSCs under hypoxic conditions for 48 h. Representative images were acquired with an inverted light microscope at 20x magnification and invasive area was calculated using ImageJ.

### 2.7. Cell Apoptosis by Flow Cytometry Analysis

One day after plating (3.5 × 10^5^ cells/well, six-well plates), MDA-MB-231 and BT-549 cells were treated with Cis-Pt (1 µM) and SLC-0111 (100 µM), alone or in combination, in hypoxic conditions and in the presence of CM-MSCs for 72 h. Then, cells were detached from culture plates with 0.02% EDTA (Invitrogen) and stained with annexin V and propidium iodide (PI) by using an annexin V-FITC apoptosis detection kit (DOJINDO) accordingly to the provider’s instruction. Cells were suspended in 500 µL of incubation buffer and analyzed by flow cytometry (BD Accuri™ C6, Ann Arbor, MI, USA).

### 2.8. Colometric Caspase-3 Activity Assay

Caspase-3 activity was determined using a colorimetric caspase-3 assay kit (ab39401, Abcam) according to the manufacturer’s protocol. MDA-MB-231 and BT-549 cells (100 × 10^3^ cells/well, six-well plates) were treated with Cis-Pt (1 µM) and SLC-0111 (100 µM), alone or in combination, under hypoxic conditions and in the presence of CM-MSCs for 72 h. A cell lysate containing 50 μg of protein was used for each analysis. The absorbance at 405 nm was measured with Multiskan FC microplate reader (Thermofisher, Waltham, MA, USA).

### 2.9. Cell Lysate Preparation and Western Blot Analysis

An equal amount of proteins from whole-cell lysates were separated by 4–12% SDS-PAGE and were transferred to a nitrocellulose membrane. Blots were blocked for 1 h with 5% non-fat dry milk and then incubated over night with the following primary antibodies: anti-CA-IX (R&D), anti-N-cadherin, anti-β-catenin and anti-Vimentin (CST-9782), anti-pro-Caspase-3, anti-Cleaved-Caspase-3, anti-AKT (CST-9272), anti-pAKT (CST-9271), anti-ERK (CST-9102), anti-pERK (CST-9101), anti-MMP-2 (CST-33437), anti-CD44 (Abcam), anti-GADPH (G8795) and anti-Actin (A4700). After washing with 0.1% Tween-20 in PBS, the filters were incubated with their respective secondary antibodies for 1 h and analyzed using the ECL system. Densitometric analyses were performed on at least two different expositions to assure the linearity of each acquisition using ImageJ software (v1.46r) [23].

### 2.10. Statistical Analysis

Data were analyzed with GraphPad Prism statistical software 8.0 (GraphPad Software, La Jolla, CA, USA). The results were obtained from at least three independent experiments and are expressed as means ± standard deviation, and significance was determined using Student’s *t*-test. A *p*-value < 0.05 was considered statistically significant.

## 3. Results

### 3.1. CM-MSCs Decrease Cis-Pt Effects and Increase CA IX Expression Levels in TNBC Cells

**Figure 1:**
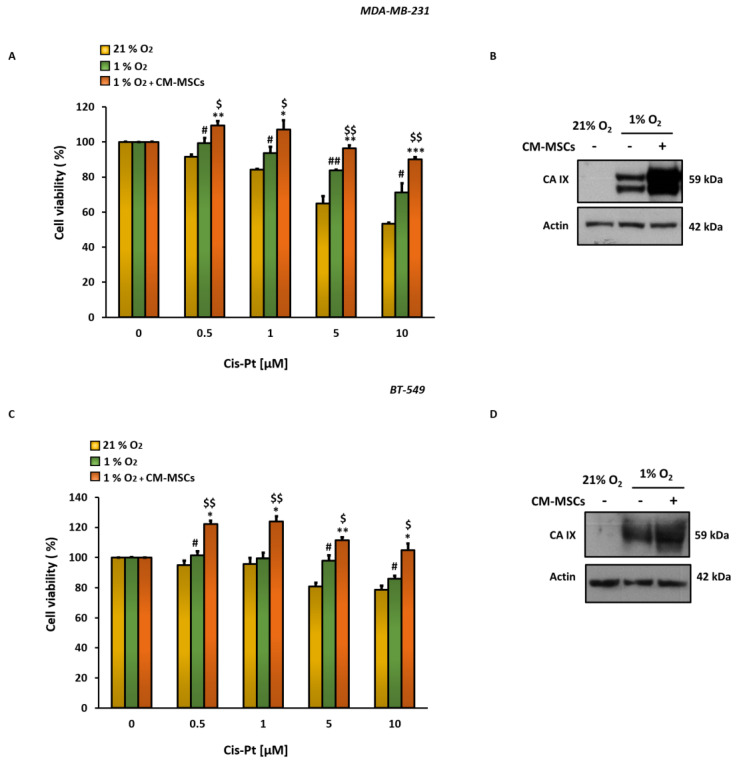
CM-MSCs in hypoxic TME increase Cis-Pt insensitivity in TNBC cells. MTS assay of MDA-MB-231 (**A**) and BT-549 (**C**) cells, grown under normoxic (21% O_2_) and hypoxic (1% O_2_) conditions in the presence or absence of CM-MSCs for 72 h and treated with different concentrations of Cis-Pt (from 0.5 µM to 10 µM). All the data are expressed as percentages of viable cells, considering the untreated control cells as 100%. Bars depict mean ± SD of three independent experiments (1% O_2_ vs. 21% O_2_: ^##^ *p* < 0.001, ^#^ *p* < 0.01; 1% O_2_ + CM-MSCs vs. 21% O_2_: *** *p* < 0.0001, ** *p* < 0.001, * *p* < 0.01; 1% O_2_ + CM-MSCs vs. 1% O_2_: ^$$^ *p* < 0.001, ^$^ *p* < 0.01). (**B**–**D**) CA IX protein levels were analyzed using Western blot analysis under the same conditions described above, and actin was used as a loading control.

In order to investigate the role of BM-MSCs in modulating the response of TNBC cells to Cis-Pt in hypoxic TME, MDA-MB-231 and BT-549 cells were grown in normoxic (21% O2) or hypoxic (1% O2) conditions in the presence or absence of CM-MSCs and treated with different concentrations of Cis-Pt (from 0.5 to 10 µM) for 72 h. The analysis of cell viability, assessed by MTS assay, showed that hypoxia causes a significant reduction of Cis-Pt effects, at all concentrations tested, with respect to normoxia in both TNBC cell lines. The addition of CM-MSCs to tumor cells grown in hypoxic conditions further reduced their sensitivity to chemotherapy (Figure 1A,C). Recently, we demonstrated the crucial role played by hypoxia-induced expression of CA IX in TNBC plasticity and aggressiveness [10]. In this context, we investigated the involvement of this enzyme in the mechanism of insensitivity induced by CM-MSCs to Cis-Pt. As expected, the TNBC cells grown in 1% O_2_ showed higher expression levels of CA IX in comparison to those grown in 21% O_2_; the presence of CM-MSCs caused further enhancement of the expression levels of this protein (Figure 1B,D).

### 3.2. Inhibition of CM-MSC-Induced CA IX by SLC-0111 Potentiates Cis-Pt Effect on TNBC Cells

**Figure 2:**
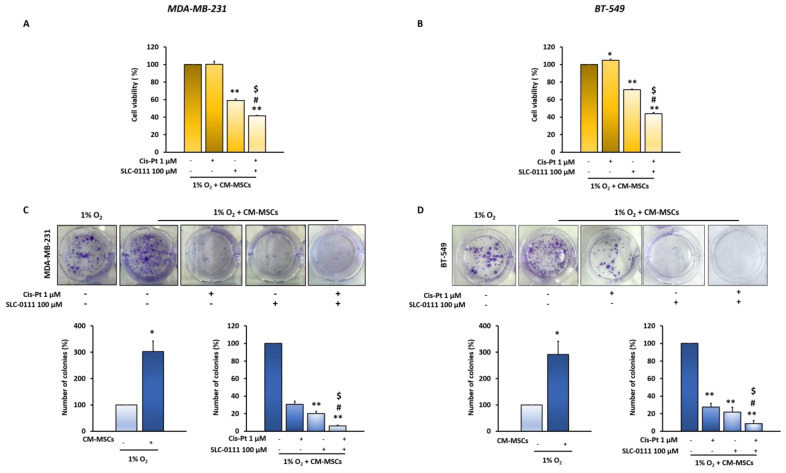
The combined treatment of SLC-0111 and Cis-Pt drastically reduces CM-MSC-induced TNBC cell viability and proliferation. Cell viability assay and clonogenic assay of MDA-MB-231 (**A**,**C**) and BT-549 (**B**,**D**) cells treated with Cis-Pt (1 µM), SLC-0111 (100 µM), alone or in combination, under hypoxic conditions (1% O_2_) and in the presence of CM-MSCs for 72 h and 10 days, respectively. All the data are expressed as percentages, considering the untreated control cells as 100%. Bars depict mean ± SD of three independent experiments (vs. untreated ** *p* < 0.0001, * *p* < 0.001; vs Cis-Pt ^$^ *p* < 0.0001; vs. SLC-0111 ^#^ *p* < 0.0001).

To assess whether the inhibition of CA IX increases TNBC cells’ response to Cis-Pt reduced by TME, MDA-MB-231 and BT-549 cells grown under hypoxic conditions in the presence of CM-MSCs were treated with the CA IX sulfonamide inhibitor SLC-0111 (100 µM), alone or in combination with low doses of Cis-Pt (1 µM), for 72 h or 10 days and then cell viability and proliferation were assessed using MTS and clonogenic assays, respectively. Notably, the combinatorial treatment with SLC-0111 and Cis-Pt caused a higher reduction of cell viability than the treatments with each drug alone (58% and 56% in MDA-MB-231 and BT-549 cell lines, respectively; *p* < 0.0001) (Figure 2A,B). Moreover, the advantage of the combinatorial treatment was recapitulated when the cells, treated in the same conditions as above, were left growing for 10 days, after which a clonogenic assay was performed. The increased duration of treatments also caused a significant reduction of TNBC clone proliferation when Cis-Pt and SLC-0111 were used as single agents. Indeed, Cis-Pt and SLC-0111 used as single agents caused a significant reduction of TNBC clone proliferation (MDA-MB-231 cells: Cis-Pt 69.4%, SLC-0111 79.7%, *p* < 0.0001; BT-549 cells: Cis-Pt 72%, SLC-0111 78%, *p* < 0.0001), which was further reduced in the presence of combinatorial treatment (MDA-MB-231 cells: 93.87%, *p* < 0.0001; BT-549 cells: 91.43%, *p* < 0.0001) (Figure 2C,D).

### 3.3. The Addition of SLC-0111 to Cis-Pt Drastically Reduces CM-MSC-Induced TNBC Cell Vascular Mimicry and Migration

**Figure 3:**
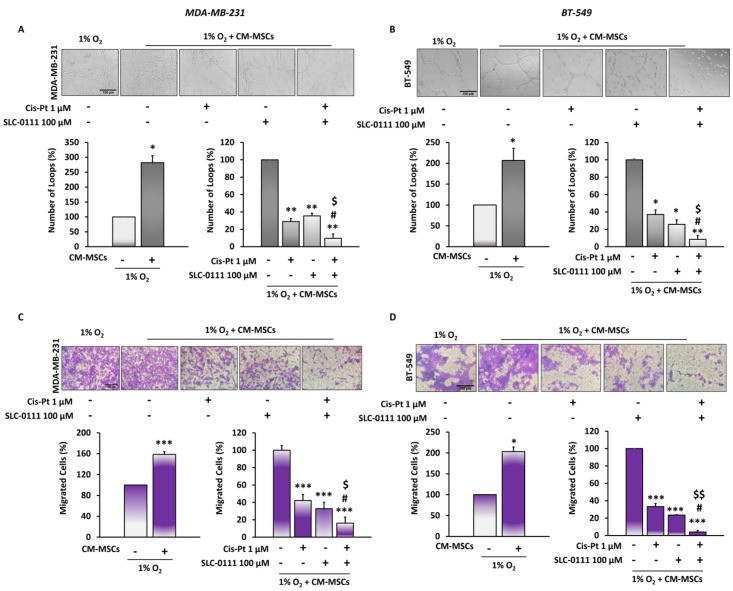
The combined treatment of SLC-0111 and Cis-Pt drastically reduces CM-MSC-induced TNBC cell migration and vessel-like structure formation. TNBC cells were grown for 24 h in hypoxia/CM-MSCs and treated with SLC-0111 (100 µM), Cis-Pt (1 µM) and a combination of the two drugs. VM of MDA-MB-231 (**A**) and BT-549 (**B**) was assessed by using 24-well plates pre-coated with 80 µL Matrigel/well and vascular loops were analyzed. Migration of MDA-MB-231 (**C**) and BT-549 (**D**) was performed by using Boyden chambers containing inserts of polycarbonate membranes with 8 μm pores. Medium containing 10% FBS was added to the lower chamber as chemoattractant. Magnification 10×, scale bar = 100 µm. All the data are expressed as percentages, considering the untreated control cells as 100%. Bars depict mean ± SD of three independent experiments (vs untreated *** *p* < 0.0001, ** *p* < 0.001, * *p* < 0.01; vs. Cis-Pt ^$$^ *p* < 0.0001, ^$^ *p* < 0.001; vs. SLC-0111 ^#^ *p* < 0.001).

The consequences of failure of chemotherapy treatment are relapse of disease and metastatic spread due to numerous effects of TME on malignancy of tumor cells. Among these, tumor cells acquire a high capacity to migrate and organize themselves into vascular-like structures. Here, we observed that CM-MSCs induced a significant increase in the formation of channel-like structures, as well as migration of both TNBC cell lines (Figure 3). In order to investigate whether the CA IX inhibitor was capable of potentiating the effect of Cis-Pt on tumor cells in an endothelial-like phenotype, TNBC cells were plated on Matrigel, grown in hypoxia in the presence of CM-MSCs and treated with SLC-0111 (100 µM), Cis-Pt (1 µM) and a combination of the two drugs for 24 h. A reduction of vessel loop formation was observed after each single treatment (MDA-MB-231 cells: Cis-Pt 71%, SLC-0111 64.6%, *p* < 0.01; BT-549 cells: Cis-Pt 62.86%, SLC-0111 74.30%, *p* < 0.01). The combined treatment of two drugs caused a further decrease of vessel loop formation (MDA-MB-231 cells: 90.3%, *p* < 0.001; BT-549 cells: 91.43%, *p* < 0.001) (Figure 3A,B).

In addition, MDA-MB-231 and BT-549 cells grown in the presence of CM-MSCs under hypoxic conditions were treated as described above and plated in the upper trans-well chambers, whereas 10% FBS was added to the lower chamber as chemoattractant. We observed that both Cis-Pt and SLC-0111 caused a significant reduction of migration of both TNBC cell lines at 24 h (*p* < 0.0001) (Figure 3C,D). The treatment of both TNBC cell lines with a combination of Cis-Pt and SLC-0111 greatly amplified the inhibitory effect on tumor cell migration (reduction of 97.3% in MDA-MB; 231 cells, *p* < 0.0001; reduction of 96% in BT-549 cells, *p* < 0.0001).

### 3.4. CA IX Inhibitor Potentiates Cis-Pt Effect on CM-MSC-Induced TNBC Cell Invasiveness

**Figure 4:**
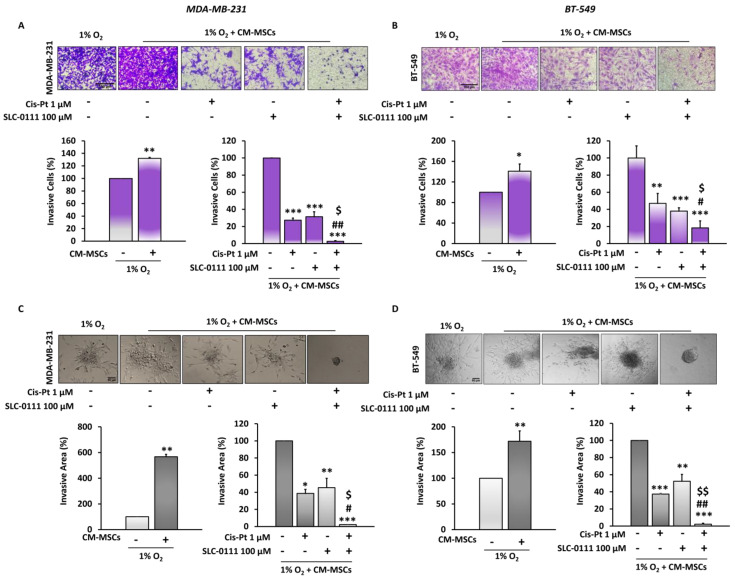
The addition of SLC-0111 to Cis-Pt decreases CM-MSC-induced TNBC cell invasiveness. Invasion of MDA-MB-231 (**A**) and BT-549 (**B**) cells in 2D, was performed by using a Boyden chamber containing inserts of polycarbonate membranes with 8 μm pores coated with Matrigel. TNBC cells were grown for 72 h under hypoxic (1% O_2_) conditions in the presence of CM-MSCs and treated with SLC-0111 (100 µM), Cis-Pt (1 µM) or a combination of the two drugs. Medium containing 10% FBS was added to the lower chamber as chemoattractant. Magnification 10×, scale bar = 100 µm. MDA-MB-231 (**C**) and BT-549 (**D**) spheroids were embedded in Matrigel and treated for 48 h as described above. Magnification 20×, scale bar = 50 µm. All the data are expressed as percentages, considering the untreated control cells as 100%. Bars depict mean ± SD of three independent experiments (vs. untreated * *p* < 0.01, ** *p* < 0.001, *** *p* ≤ 0.0001; vs. Cis-Pt ^$$^ *p* < 0.0001, ^$^ *p* < 0.001; vs. SLC-0111 ^#^ *p* < 0.001, ^##^ *p* ≤ 0.0001).

In order to analyze the effects of CM-MSCs on TNBC invasiveness, we performed 2D and 3D invasion assays using trans-well chambers coated with Matrigel and spheroids embedded in Matrigel, respectively. The experiments were carried out in the presence of CM-MSCs under hypoxic conditions. Firstly, we observed that in both TNBC cell lines, CM-MSCs increased their ability to invade the extracellular matrix in 2D at 72 h. The treatment of tumor cells with Cis-Pt (1 µM) and SLC-0111 (100 µM) as single agents significantly reduced TNBC cell invasiveness (MDA-MB-231 cells: Cis-Pt 58%, SLC-0111 67%; BT-549 cells: Cis-Pt 53%, SLC-0111 62%). The addition of SLC-0111 to Cis-Pt greatly amplified its inhibitory effect on tumor cell invasion (MDA-MB-231 cells: 84%; BT-549 cells: 82%) (Figure 4A,B). Next, to evaluate the capability of the tumor cells organized into a 3D structure (mimicking a tumor micro-region with stemness features) to invade the matrix, spheroids obtained from both TNBC cell lines were embedded in Matrigel and grown in the presence of CM-MSCs under hypoxic conditions. After 72 h, they showed numerous and long protrusions invading Matrigel that were strongly reduced by treatment with both Cis-Pt and SLC-0111 (MDA-MB-231 cells: Cis-Pt 62%, SLC-0111 55%; BT-549 cells: Cis-Pt 63%, SLC-0111 48%). Notably, the combination of the two drugs completely abolished the invasiveness capability of the spheroids (MDA-MB-231 cells: 97.67%; BT-549 cells: 97.70%) (Figure 4C,D).

### 3.5. CA IX Inhibition Increases Cis-Pt Effects in Inducing Apoptosis in TNBC Cells

**Figure 5:**
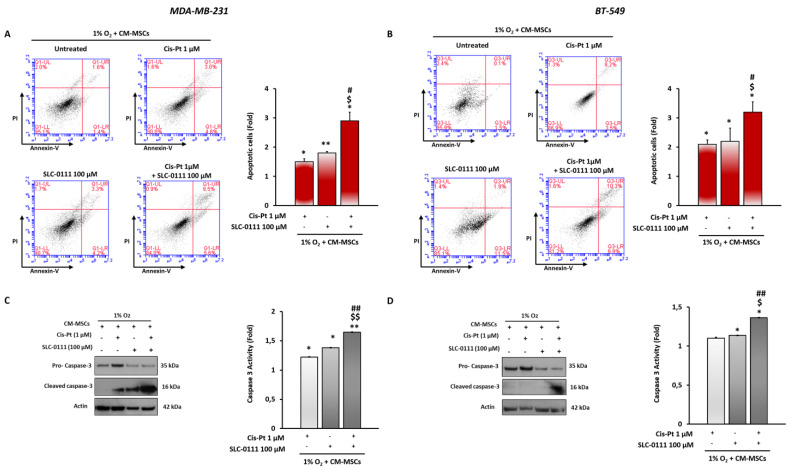
SLC-0111 potentiates the effect of Cis-Pt in increasing apoptotic programs in TNBC cells. MDA-MB-231 (**A**) and BT-549 (**B**) cells grown for 48 h in the presence of CM-MSCs and hypoxia (1% O_2_) and treated with Cis-Pt (1 µM), SLC-0111 (100 μM) and a combination of the two drugs were stained with annexin V/PI and subjected to flow cytometry analysis. Analysis of expression levels of the active cleaved form of caspase-3 and its enzymatic activity were performed using Western blotting and colorimetric assay, respectively, in MDA-MB-231 (**C**) and BT-549 (**D**) cells. Equal loading was confirmed by actin. Representative images are shown. Values are shown relative to untreated cells, arbitrarily set to 1 (*n* = 3). Bars depict mean ± SD of three independent experiments. (vs. untreated ** *p* < 0.01, * *p* < 0.1; vs. Cis-Pt ^$$^ *p* < 0.001, ^$^ *p* < 0.01; vs SLC-0111 ^##^ *p* < 0.001, ^#^ *p* < 0.01).

Next, we evaluated whether CA IX inhibition increases Cis-Pt effect in inducing apoptosis when TNBC cells were grown in the presence of CM-MSCs and hypoxia. Cell death was measured using flow cytometry after staining MDA-MB-231 and BT-549 cells with annexin V/PI. As shown in Figure 5A,B, the combined treatment with the two drugs significantly increased the number of apoptotic cells with respect to treatment with single agents. To confirm the ability of SLC-0111 to enhance the effect of Cis-Pt on activation of an apoptotic program in the presence of CM-MSCs, the expression levels and enzymatic activity of apoptosis effector caspase 3 were analyzed. Combination treatment with the two drugs showed, compared with single treatments, a greater reduction in full-length pro-caspase-3 and a strong increase in its cleaved active form, as assessed by Western blotting, and an enhancement of caspase-3 enzymatic activity, as analyzed by colorimetric assay (Figure 5C,D).

### 3.6. SLC-0111 Increases Cis-Pt Effect on CM-MSC-Induced Signaling Pathways in TNBC Cells

**Figure 6:**
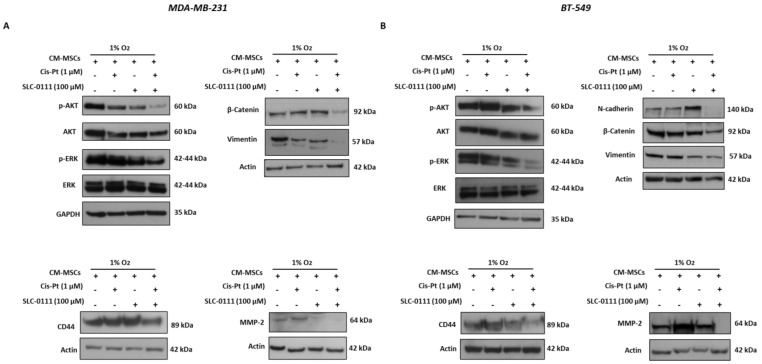
SLC-0111 increases Cis-Pt effect on CM-MSC-induced signaling pathways in TNBC cells. MDA-MB-231 (**A**) and BT-549 (**B**) cells grown for 72 h in hypoxic conditions (1% O_2_) and in presence of the CM-MSCs were treated with SLC-0111 (100 µM) and Cis-Pt (1 µM), alone or in combination. Expression levels of p-ERK/ERK, p-AKT/AKT, N-cadherin, β-Catenin, vimentin, CD44 and MMP-2 were analyzed. Equal loading was confirmed by immunoblot with anti-actin and anti-GADPH antibodies. Representative images are shown.

Signaling pathways underlying the mechanisms associated with the limited response to chemotherapy due to the presence of BM-MSCs in the hypoxic TME were also investigated. Combinatorial treatment of both TNBC cell lines grown in the presence of CM-MSCs/hypoxia with Cis-Pt (1 µM) and SLC-0111 (100 µM) reduced the expression levels of phosphorylated forms of AKT and ERK to a greater extent than observed in cells treated with the single agents (Figure 6A,B).

Next, we analyzed whether proteins that play key roles in the EMT program and stemness, which are closely interconnected with each other and with numerous events associated with chemoresistance, are involved in the strong inhibition of cancer cell migration and invasiveness observed after the combinatorial therapeutic approach. As shown in Figure 6A,B, the expression levels of mesenchymal markers such as β-catenin and vimentin and N-cadherin, expressed only in BT-549 cells, and the stemness marker CD44 were dramatically reduced when SLC-0111 was added to Cis-Pt. Finally, the expression levels of the matrix-degrading enzyme metalloproteinase-2 (MMP-2), one of the promoters of tumor cell invasion, were also found to be more reduced after combined treatment with the two drugs compared with the single agents.

## 4. Discussion

Conventional chemotherapy represents the main systemic treatment used for most TNBC patients in both first-line settings and advanced stages of the disease, although many patients develop drug resistance [24,25]. The insensitivity to chemotherapy is associated with a 40 to 80% risk of recurrence, resulting in distant metastasis and death for most patients [26]. Reverting resistance, as well as improving sensitivity to chemotherapy, is a critical unmet clinical need in TNBC [2].

To better understand how to improve the efficacy of anticancer-therapies, the contributions of TME components in mediating cancer cell resistance to therapeutic interventions must be encompassed. The most apparent function of tumor stroma is to provide a structural support to tumor cells, but it has become increasingly evident that it also interacts with tumor cells and evolves with them [27]. Cross-talk between stromal cells and tumor cells influences the behavior of both by promoting tumor progression and aggressiveness in terms of metastasis, disease relapse and failure of therapy [8,28]. Many studies have shed light on the involvement of mesenchymal stem cells in various aspects of breast cancer pathogenesis, including resistance to therapies [8]. In addition, it is well known that hypoxia and MSCs are the crucial drivers in the onset of insensitivity to chemotherapy through multiple mechanisms [8,29]. Chen et al. showed that conditioned medium collected from adipose-derived MSCs enhanced breast cancer resistance protein (BCRP) expression, leading to reduced doxorubicin (Dox) sensitivity in TNBC cells [30]. Similar investigations found that the exosome secreted by Dox-treated MSCs induced a higher degree of Dox resistance in breast cancer cells via miR-21-5p [31]. Furthermore, recent studies carried out in breast cancer cells and murine models demonstrated the involvement of MSCs in promoting resistance to trastuzumab through induction of lncRNAAGAP2-AS1 [32] and to estrogen-targeted therapies by iron modulation and CSC transition [33].

Although Cis-Pt is not considered a standard of care for TNBC, many clinical trials are evaluating its use as single agent or in combination with other therapies [2,34,35] and several strategies are under investigation to improve its efficacy while reducing toxicity [36].

Much evidence has reported the involvement of hypoxic TME in the onset of Cis-Pt resistance in cancer patients [37]. In addition, several findings have shown the crucial role played by MSCs in reducing Cis-Pt effects in breast cancer [38] and nasopharyngeal carcinoma [39]. Our previous studies demonstrated the ability of medium conditioned from these stromal cells to increase aggressiveness of osteosarcoma and hepatocellular carcinoma cells through CXCR4 and AQP1 [40,41]. Notably, we showed BM-MSCs homing into TNBC TME, their trans-differentiation in cancer-associated fibroblasts with high levels of α-SMA, FAP and FSP-1 when co-cultured with TNBC cells and, consequently, their support in metastatic spread of tumor cells [9]. The use of a specific anti-PDGFR-β aptamer blocked BM-MSCs’ pro-metastatic effect on TNBC in vitro and in vivo [8,42]. In more recent studies, we clarified the effects of hypoxia on TNBC plasticity and HNSCC Cis-Pt resistance through CA IX overexpression [10,43]. Interestingly, Ong et al. demonstrated that CA IX expression is independently associated with a poorer clinical and survival outcome in TNBC patients [44].

Therefore, in this study, we investigated whether this enzyme could be involved in the occurrence of the reduced chemotherapy sensitivity of TNBC cells induced by BM-MSCs. We found that these stromal cells under hypoxic conditions caused a greater increase in CA IX expression levels and Cis-Pt insensitivity in TNBC cells than in the same cells grown and treated under hypoxic conditions alone. Here, we used a novel CA IX inhibitor, SLC-0111, to revert this BM-MSC-induced Cis-Pt resistance. This molecule has been reported to sensitize tumor cells to several chemotherapeutic treatments, such as melanoma cells to decarbazine and temozolomide (TMZ), breast cancer cells to Dox and colorectal cancer cells to 5-fluoracil [45], glioblastoma patient-derived xenografts to TMZ [46], KRAS-driven pancreatic ductal adenocarcinoma xenografts to gemcitabine [47] and HNSCC cells and xenografts to Cis-Pt [43]. Our results showed that BM-MSCs under hypoxic conditions augment the ability of TNBC cells to form channel-like formations on Matrigel, a hallmark of VM, as well as the ability to migrate and invade. We also found that the addition of the CA IX inhibitor to Cis-Pt was able to block these mechanisms more effectively than treatment with single agents. Furthermore, we showed that the combined treatment hindered the signaling pathways underlying these malignant processes more than Cis-Pt and SLC-0111 alone. In fact, a greater reduction in the expression levels of phosphorylated forms of AKT and ERK, as well as the stemness marker CD44 and the invasion marker MMP-2, was observed. Interestingly, the aggressive mesenchymal phenotype was also prevented, as evidenced by reduced expression levels of vimentin, β-catenin and N-cadherin. The effect of CA IX inhibition to improve Cis-Pt efficacy was confirmed by a significant enhancement of the apoptotic program compared with single treatments, as assessed by annexin V assay, caspase-3 activity and expression of the cleaved active form of caspase-3.

## 5. Conclusions

A major barrier to successful chemotherapy treatment of TNBC is due to a complex hypoxic TME, comprising different stromal cells, such as BM-MSCs. This results in poor patient prognosis, treatment resistance and metastasis. In this study, we shed light on how the presence of BM-MSCs in the TME further increases hypoxia-induced Cis-Pt insensitivity and CA IX overexpression in TNBC cells. Overall, our results showed that BM-MSCs/hypoxia-induced reduction of Cis-Pt sensitivity can return to a chemosensitive state through CA IX inhibition in TNBC cells. Further studies in a syngeneic TNBC murine model with intact microenvironment will allow us to confirm the ability of SLC-0111 to potentiate the response to cisplatin in vivo.

## Data Availability

Not applicable.
